# Influenza vaccination coverage and determinants of vaccination among older adults in Turkey

**DOI:** 10.1017/S0950268826101563

**Published:** 2026-04-30

**Authors:** Buşra Tozduman, Buğra Taygun Gülle

**Affiliations:** 1 Public Health, Dokuz Eylul University Faculty of Medicine, Türkiye; 2 Izmir Provincial Directorate of Health, Türkiye

**Keywords:** health determinants, influenza vaccination, older adults, Turkey, vaccination coverage

## Abstract

This study aimed to identify determinants of influenza vaccination among older adults using nationally representative data from the Turkey Older Persons Profile Survey 2023. Data from 11 657 individuals aged 65 and over, collected by the Turkish Statistical Institute, were analysed. Least Absolute Shrinkage and Selection Operator regression was employed for variable selection, followed by binary logistic regression to identify significant predictors. Only 19.4% of older adults reported receiving an influenza vaccine during the 2022/2023 influenza season. Higher education, income sufficiency, social security coverage, regular medication use, physical activity, and use of mobile health (mHealth) applications were significantly associated with higher vaccination uptake. Former smoking, alcohol consumption, older age, higher body mass index, and greater independence in daily living were also positive predictors. Traditional barriers to healthcare access (e.g., transportation, waiting times) were not significantly associated. Regional disparities were evident, with lower vaccination rates in the eastern regions. Vaccine uptake among older adults in Turkey is low despite universal access. Promoting engagement with primary healthcare services and increasing the use of mHealth applications may contribute to increasing vaccination coverage. Special attention should be given to socially disadvantaged groups and underperforming regions to enhance preventive healthcare among the aging population.

## Introduction

Seasonal influenza impacts nearly one billion people worldwide each year, leading to an estimated three to five million cases of severe illness and causing between 290 000 and 650 000 deaths due to respiratory complications. Those at increased risk for severe outcomes include pregnant women, young children under five, older adults, and individuals with chronic health conditions – such as cardiovascular, pulmonary, renal, metabolic, neurodevelopmental, hepatic, or haematological disorders – as well as individuals with immunosuppressive conditions or undergoing immunosuppressive treatments (e.g., HIV infection, chemotherapy, steroid therapy, or malignancy). The virus spreads easily via respiratory droplets expelled when an infected person coughs or sneezes. Vaccination continues to be the most effective method for preventing influenza and its associated complications [1].

According to data from the Turkish Statistical Institute (TurkStat), 10.6% of the Turkish population consisted of people aged 65 and above in 2024. Based on the main scenario of population projections – which assumes that current demographic trends will continue – the proportion of older adults is expected to reach 13.5% by 2030 and 17.9% by 2040 [2]. In Turkey, influenza vaccination is provided free of charge for this age group. Nevertheless, previous studies have shown that influenza vaccine uptake remains low among older adults in the country [3–13]. Identifying the determinants of vaccine uptake in this population is crucial for informing targeted interventions and improving vaccination coverage. However, most existing studies on this topic in Turkey have been single-centre investigations, lacking national representativeness.

This study aimed to identify the determinants of influenza vaccination among older adults in Turkey using nationally representative data from the Turkey Older Persons Profile Survey 2023, conducted by TurkStat. By examining a comprehensive set of sociodemographic, health-related, and healthcare access variables, this research seeks to inform evidence-based public health strategies to enhance vaccine uptake among the ageing population.

## Material–method

This study utilized data from the 2023 Turkey Older Persons Profile Survey conducted by the TurkStat [14]. The survey collected comprehensive information on individuals aged 50 and above, including demographic characteristics, employment and economic conditions, health status, independent living, care and social assistance, environmental factors, participation in social life, life satisfaction, disaster and emergency awareness and preparedness, and perceptions of rights and discrimination. It also included household-level data such as education level, household income, housing conditions, and general environmental information [14].

A total of 22 640 households, each containing at least one person aged 50 or above, were selected as the sample [14]. From these households, data from 11 657 individuals aged 65 and over (5 232 men and 6 425 women) were used in this study.

Variables potentially associated with influenza vaccination status included age, sex, marital status, education level, living alone, employment status, income sufficiency, presence of social security coverage, body mass index (BMI), general health status, presence of chronic conditions, officially documented disability, regular use of prescribed medications, and the first point of contact in the healthcare system.

Health behaviour-related factors included tobacco and alcohol consumption, physical activity, use of mobile health (mHealth) applications, and receipt of home healthcare services.

Regional differences were also considered based on place of residence, according to the Nomenclature of Territorial Units for Statistics (NUTS-1) classification.

In addition, participants’ difficulties in accessing healthcare – such as transportation to health institutions, communication with healthcare professionals, making an appointment, accessing a preferred physician, doing paperwork, waiting in line, medication-related procedures, and physical conditions of healthcare facilities – were included.

Functional status was assessed using the Washington Group Short Set on Functioning, the Lawton–Brody Instrumental Activities of Daily Living Scale, and the Katz Index of Independence in Activities of Daily Living.

**Figure 1.**
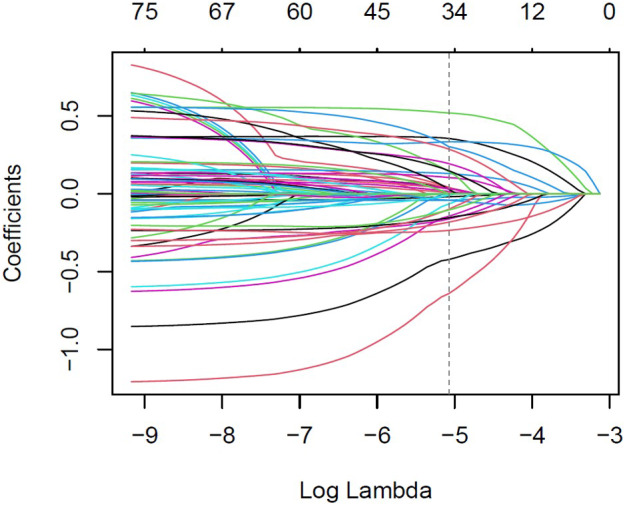
LASSO regression curve-coefficient versus log (*λ).*

**Figure 2.**
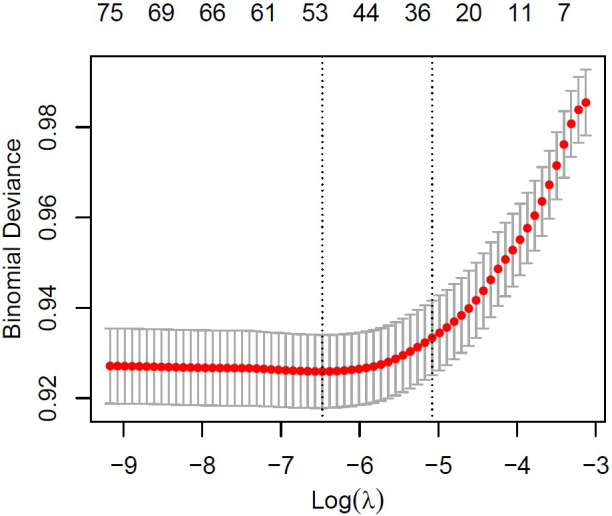
LASSO regression curve-binomial deviance versus log (*λ).*

To determine the variables most significantly associated with influenza vaccination, the Least Absolute Shrinkage and Selection Operator (LASSO) regression was applied. This method was particularly suitable due to the high number of candidate predictors and the risk of multicollinearity among them. Figures 1 and 2 illustrate the variable selection process using LASSO. The penalty parameter lambda (*λ*) was selected based on 10-fold cross-validation, and binomial deviance was computed to evaluate the model’s predictive accuracy on the test data.

In Figure 1, each line traces the trajectory of a single predictor’s coefficient across varying values of *λ.* The dashed vertical line indicates the optimal *λ.* Figure 2 displays red points representing the cross-validated estimates of binomial deviance for each *λ* value. Two vertical dashed lines indicate: (i) the *λ* that achieves the minimum deviance, and (ii) the largest *λ* within one standard error of that minimum. The second *λ* was selected, as it favoured a more simplified model while maintaining adequate predictive performance.

A total of 21 variables were selected at *λ* = 0.006231793, including age, BMI, Lawton–Brody scale, marital status, education level, employment status, income sufficiency, presence of social security coverage, general health status, presence of chronic disease, regular use of prescribed medications, first consulted health institution, difficulty accessing a preferred physician, waiting time, limited examination periods, physical conditions of the healthcare institution, tobacco use, alcohol use, physical activity level, use of mobile health applications, and region.

These preliminary variables were then entered into a binary logistic regression model for further analysis. All statistical tests were two-sided. Data analyses and visualizations were conducted using R version 4.3.1 (R Foundation for Statistical Computing, Vienna, Austria).

## Results

A total of 11 657 older adults aged 65 years and above were included in the study. Of the participants, 55.1% (*n* = 6 425) were female. The majority were aged 65–69 years (37.8%, *n* = 4 412), followed by 70–74 years (26.7%, *n* = 3 108) and 75–79 years (17.1%, *n* = 1 997). Regarding marital status, 64.3% (*n* = 7 490) were married and 31.8% (*n* = 3 704) were widowed. In terms of educational attainment, 24.1% (*n* = 2 808) were illiterate, and 45.1% (*n* = 5 262) had completed primary school. Approximately 19.6% (*n* = 2 288) of the participants lived alone. Most of the participants were either retired (38.6%, *n* = 4 494) or engaged in domestic work or caregiving duties (32.6%, *n* = 3 804). Most participants rated income sufficiency as either ‘moderate’ (26.8%, *n* = 3 120) or ‘hard’ (25.1%, *n* = 2 930). About 13.8% (*n* = 1 611) of the participants had no social security coverage. By design, the highest proportions of participants were sampled from the Aegean Region (13.3%, *n* = 1 552), the Mediterranean Region (10.7%, *n* = 1 247), and Istanbul (10.3%, *n* = 1 203), whereas the lowest came from Northeastern Anatolia (4.3%, *n* = 503) and Central Eastern Anatolia (5.9%, *n* = 689) (Table 1).

**Table 1.** Sociodemographic characteristics of the participants

		*N*	%
Sex	Female	6 425	55.1
	Male	5 232	44.9
Age group	65–69	4 412	37.8
	70–74	3 108	26.7
	75–79	1 997	17.1
	80–84	1 237	10.6
	85–89	562	4.8
	90–94	292	2.5
	95+	49	0.4
Marital status	Never married	136	1.2
	Married	7 490	64.3
	Divorced	326	2.8
	Widowed	3 704	31.8
Education	Illiterate	2 808	24.1
	No school completed	1 091	9.4
	Primary school	5 262	45.1
	General secondary school/vocational or technical secondary school/primary education	659	5.7
	General high school/ vocational or technical high school	912	7.8
	2 or 3 years higher educational institutions/4 years universities or other higher educational institutions	834	7.1
	5 or 6 years faculties/Master (Except for 5 or 6 years faculties)/Doctorate	91	0.8
Living alone	Yes	2 288	19.6
Employment status	Employed	1 298	11.1
	Not retired nor employed	1 554	13.4
	Retired or left work life due to reasons related to age	4 494	38.6
	Disabled and/or unable to work due to permanent health problems	506	4.3
	Occupied with housework and/or with the care of children, elderly, ill, etc., people in the family	3 804	32.6
Income sufficiency	Very hard	1 091	9.4
	Hard	2 930	25.1
	Moderate	3 120	26.8
	Easy	1 110	9.5
	Very easy	119	1.0
Have social security coverage	No	1 611	13.8
Region	TR1: İstanbul	1 203	10.3
	TR2: West Marmara	893	7.7
	TR3: Aegean	1 552	13.3
	TR4: East Marmara	1 165	10.0
Region	TR5: West Anatolia	1 050	9.0
	TR6: Mediterranean	1 247	10.7
	TR7: Central Anatolia	757	6.5
	TR8: West Black Sea	1 086	9.3
	TR9: East Black Sea	765	6.6
	TRA: North East Anatolia	503	4.3
	TRB: Central East Anatolia	689	5.9
	TRC: South East Anatolia	747	6.4

Among the participants, 48.7% (*n* = 5 673) rated their general health status as ‘moderate’, while 25.2% (*n* = 2 936) rated it as ‘bad’. A high percentage (78.8%, *n* = 9 184) reported having at least one chronic condition. Officially documented disability was reported by 8.7% (*n* = 1 019) of participants. Additionally, 79.7% (*n* = 9 288) were regularly taking prescribed medications. Approximately two-thirds of participants were either overweight (40.8%, *n* = 4 760) or obese (27.0%, *n* = 3 146). Daily tobacco use was reported by 11.2% (*n* = 1 300), while 21.3% (*n* = 2 479) were former smokers. Only 11.4% (*n* = 1 327) reported ever consuming alcohol. Regarding physical activity, 52.4% (*n* = 6 111) indicated that they never exercised, whereas 17.4% (*n* = 2 026) reported engaging in physical activity every day or almost every day. The proportion of participants who had received an influenza vaccination within the past year was 19.4% (*n* = 2 266). According to the Washington Group Short Set, 41.5% (*n* = 4 834) had at least one functional limitation. The mean score on the Lawton–Brody Instrumental Activities of Daily Living Scale was 5.72, and the mean Katz Index score was 5.4 (Table 2).

**Table 2.** Health-related characteristics of the participants

		*N*	%
General health status	Very good	146	1.3
	Good	2 426	20.8
	Moderate	5 673	48.7
	Bad	2 936	25.2
	Very bad	476	4.1
Chronic disease	Yes	9 184	78.8
Officially documented disability	Yes	1 019	8.7
Regular prescribed medication use	Yes	9 288	79.7
BMI	Underweight (<18.5)	174	1.5
	Healthy weight (18.5–24.9)	3 577	30.7
	Overweight (25–29.9)	4 760	40.8
	Class 1 Obesity (30–34.9)	2 256	19.4
	Class 2 Obesity (35–39.9)	679	5.8
	Class 3 Obesity (≥40)	211	1.8
Tobacco consumption	Yes, every day	1 300	11.2
	Yes, occasionally	245	2.1
	No, I used to smoke	2 479	21.3
	No, never smoked	7 633	65.5
Ever consuming alcohol	Yes	1 327	11.4
Alcohol use frequency in the past 12 months	Daily or almost daily	62	4.6
	1–2 days per week	114	8.6
	2–3 days per month	107	8.1
	About once a month	231	17.4
	Former drinker	813	61.3
Physical activity	Never	6 111	52.4
	Rarely	1 958	16.8
	1–3 times a month	389	3.3
	At least once a week	1 173	10.1
	Almost every day	2 026	17.4
Influenza vaccination	Yes	2 266	19.4
The Washington group is short set on functioning	At least one functional limitation	4 834	41.5
		mean ± S.D.	
Lawton–Brody instrumental activities of daily living scale		5.72 ± 2.44	
Katz’s index of independence in activities of daily living		5.4 ± 1.44	

Regarding the first point of contact in the healthcare system, 46.9% (*n* = 5 464) first consulted a public hospital, while 43.6% (*n* = 5 085) visited a family health centre. Approximately 32.1% (*n* = 3 740) of participants reported difficulties with transportation to healthcare facilities, and 49.6% (*n* = 5 777) experienced challenges making an appointment. Other common barriers included difficulty accessing the doctor of choice (29.8%, *n* = 3 471) and waiting in line (31.7%, *n* = 3 696). Use of mobile health applications, such as the Centralized Appointment System (MHRS) and the Personal Health Record System (e-Nabız), was reported by 13.4% (*n* = 1 565) of participants. Additionally, 3.3% (*n* = 379) had received home healthcare services within the last 12 months (Table 3). Distributions by vaccination status are also provided in Supplementary Table S1.

**Table 3.** Characteristics of healthcare utilization and access barriers

		*N*	%
First consulted health institution	Family health centre	5 085	43.6
	Public hospital	5 464	46.9
	University hospital	277	2.4
	Private hospital	485	4.2
	Private polyclinic/medical centre	13	0.1
	Private clinic	11	0.1
	City hospital	291	2.5
	Traditional methods/complementary medicine (cupping, folk healer, reiki, etc.)	9	0.1
	Home healthcare	22	0.2
Have difficulties with	Transportation to a health institution	3 740	32.1
	Communication with health professionals	2 002	17.2
	Making an appointment	5 777	49.6
	Accessing a preferred physician	3 471	29.8
	Doing paperwork during healthcare	2 771	23.8
	Waiting in line	3 696	31.7
	Limited examination periods	2 795	24.0
	The process of taking medication	1 334	11.4
	Physical conditions of the health institution	1 920	16.5
Received home healthcare in the last 12 months?	Yes	379	3.3
mHealth usage	Yes	1 565	13.4

**Figure 3.**
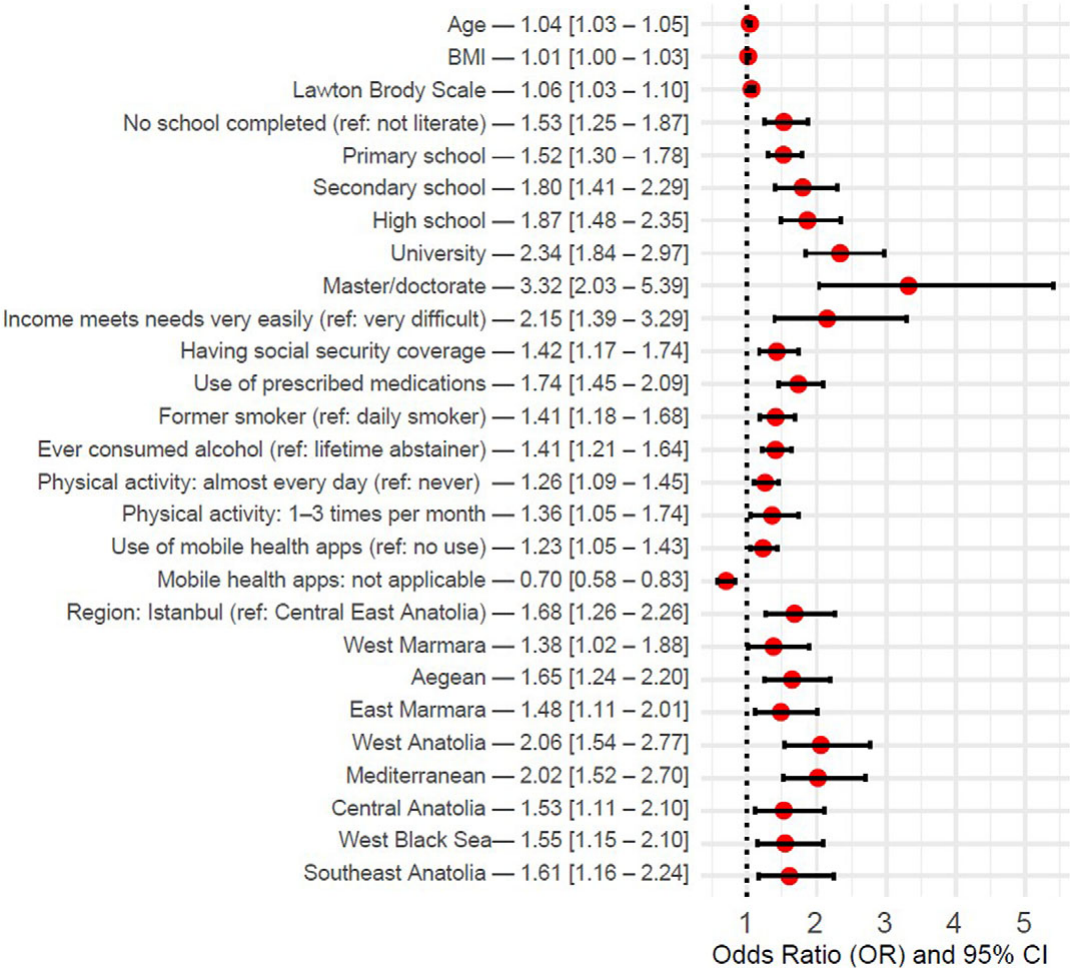
Determinants for influenza vaccination – binary logistic regression results.

Figure 3 presents the results of the logistic regression model constructed using the 21 variables selected through LASSO regression. Only variables that were significantly associated with influenza vaccination uptake were included in the graph. The strongest positive predictor of vaccination was a higher level of education, particularly at the master’s or doctorate level (OR: 3.32, 95% CI: 2.03–5.39). Vaccination was also more likely among individuals whose income met their needs very easily (OR: 2.15, 95% CI: 1.39–3.29), those with social security coverage (OR: 1.42, 95% CI: 1.17–1.74), those who regularly used prescribed medications (OR: 1.74, 95% CI: 1.45–2.09), former smokers (OR: 1.41, 95% CI: 1.18–1.68), and individuals who had ever consumed alcohol (OR: 1.41, 95% CI: 1.21–1.64). Higher physical activity (OR: 1.26, 95% CI: 1.09–1.45 for almost daily activity) and mHealth apps usage (OR: 1.23, 95% CI: 1.05–1.43) were also positively associated with vaccination. Increases in age (OR: 1.04, 95% CI: 1.03–1.05), BMI (OR: 1.01, 95% CI: 1.00–1.03), and Lawton–Brody scores (OR: 1.06, 95% CI: 1.03–1.10) were positively associated with vaccination as well.

Additionally, geographical region influenced vaccination status, with higher uptake observed particularly in West Anatolia (OR: 2.06, 95% CI: 1.54–2.77) and the Mediterranean region (OR: 2.02, 95% CI: 1.52–2.70) compared with Central East Anatolia. Other variables included in the model – such as marital status, employment status, general health status, presence of chronic conditions, first point of contact in the healthcare system, difficulty accessing a preferred physician, waiting in line, limited examination periods, and physical conditions of the hospital – were not significantly associated with influenza vaccination (see Supplementary Table 2).

## Discussion

In this study, 19.4% of the individuals aged 65 and over reported receiving an influenza vaccination within the past year. Older age, higher educational attainment, sufficient income to meet basic needs, and having social security coverage were positively associated with vaccination uptake. Individuals who reported using prescribed medications, had a higher BMI, maintained independence in activities of daily living, and used mHealth applications were also more likely to be vaccinated. Vaccination rates were additionally higher among former smokers, those who had ever consumed alcohol, and participants with higher levels of physical activity. Regional differences in vaccination uptake were observed, with higher rates particularly in Western Anatolia and the southern regions of Turkey.

In previous studies conducted in Turkey, influenza vaccination rates among individuals aged 65 and over have varied between 9.2% and 41.3% [3–13]. Most of these studies were single-centre. Moreover, the question used to assess vaccination status also differed across studies – for example, some asked whether participants had ever received an influenza vaccine, while others focused on vaccination within the past year. Although coverage rates differed across studies, nearly all reported influenza vaccination uptake among older adults to be far below the World Health Organization’s recommended target of 75%.

The observed positive association between increasing age and higher vaccination rates aligns with the literature [8, 15–17]. Older adults may have more frequent contact with healthcare providers, which increases the likelihood of receiving a physician’s recommendation – an important factor that has been shown to significantly improve seasonal influenza vaccine uptake [3]. In a study conducted in Turkey, all vaccinated individuals aged 65 and over reported that their decision to receive the influenza vaccine was based on their physician’s advice [18]. Indeed, a recent review of influenza immunization policies and implementation in the Eastern Mediterranean Region identified lack of knowledge as the most significant barrier to vaccination [19]. Similarly, a study from Turkey demonstrated that individuals with better knowledge about influenza were more likely to be vaccinated [12]. These findings suggest that physician engagement may contribute to vaccination both directly, through recommendation, and indirectly, by enhancing patient knowledge.

Higher socioeconomic status, as proxied by higher levels of education and income, was associated with higher influenza vaccination rates, consistent with previous studies [5, 13, 15–17]. A study examining awareness of influenza and pneumococcal vaccines also reported significantly greater awareness among individuals with higher levels of education [20]. In our study, the association between education level and vaccination appeared to follow a more linear pattern, whereas the impact of income was only evident at the extremes: individuals who reported meeting their basic needs ‘very easily’ were significantly more likely to be vaccinated compared to those who reported doing so ‘very difficultly’. This suggests that, in the Turkish context, income may play a comparatively less prominent role than education. Yet, these findings highlight how socioeconomic disparities can influence health behaviours. Individuals with lower socioeconomic status often exhibit less favourable health behaviours and consequently experience poorer health outcomes. In this context, having health insurance coverage also emerged as a significant factor. Notably, although all Turkish citizens residing in Turkey were brought under the General Health Insurance scheme in 2012, our findings suggest that disparities in access or coverage may still exist [21].

The presence of chronic conditions has been identified as a determinant of influenza vaccination in numerous studies [5, 8, 10, 13, 15–17]. Another study reported that influenza and pneumococcal vaccine awareness was higher among individuals aged 65 and over who had comorbidities [20]. However, in our analysis, while having a chronic illness was not significantly associated with vaccination in the multivariable model, prescribed medication use was. This may suggest that individuals on regular medications have more frequent interactions with healthcare services, which could facilitate vaccine uptake. Similarly, the observed association between higher BMI and vaccination may also reflect greater engagement with the healthcare system. A meta-analysis examining the relationship between obesity and influenza/pneumococcal vaccination also found that adults with obesity were more likely than their non-obese counterparts to receive these vaccines. The study suggested that this association may be partly explained by more frequent counselling on obesity-related risks, which could increase perceived vulnerability among individuals with obesity [22].

Variables related to access to healthcare were not found to be significant in the multivariable analysis in our study. However, higher vaccination rates were observed among individuals who were functionally independent in daily life. Similarly, the study by Gürsoy et al. also reported higher vaccination rates among those who were able to meet their own physical needs [13]. In addition, the use of mHealth applications was associated with a greater likelihood of being vaccinated. In Turkey, a commonly used mHealth application is ‘e-Nabız’, developed by the Ministry of Health. This platform provides influenza vaccination reminders and also allows users to schedule appointments, which may help increase vaccine uptake among older adults. These results indicate that, rather than structural access issues, personal autonomy and proactive health behaviours may be more critical in promoting vaccination among older adults in Turkey.

In our study, lifestyle-related factors such as smoking, alcohol consumption, and physical activity were significantly associated with influenza vaccination uptake, consistent with previous literature [15–17]. Health motivation and health consciousness have also been shown to influence engagement in preventive healthcare behaviours [23]. The higher vaccination rates observed among former smokers in this study may reflect a shift towards healthier lifestyles or a heightened perception of health risks, prompting greater use of preventive services. In Turkey, alcohol consumption is typically more prevalent among individuals of higher socioeconomic status, which may partly explain the higher vaccination rates among those who reported ever consuming alcohol [24]. Additionally, due to the overall low frequency of alcohol use, this variable was analysed as a binary indicator (ever consumed alcohol). Notably, 61% of ever consumers reported that they no longer drink. Moreover, given that both alcohol and tobacco use are socially and religiously disapproved of in some communities in Turkey, it is possible that vaccine hesitancy may also be more pronounced within these groups.

Regional disparities in vaccination uptake, considered alongside the finding that access to healthcare services was not a significant factor in multivariable analyses, suggest that unmeasured factors – such as regional differences in health literacy, vaccine-related knowledge and attitudes – may have played an influential role. In a nationally representative study conducted by the Turkish Ministry of Health in 2023, health literacy levels were found to vary significantly across regions, with TR2 (West Marmara) and TR3 (Aegean) regions exhibiting higher levels of health literacy compared to other parts of the country [24]. Although overall health literacy has improved compared to the 2017 levels – likely as a result of national health literacy promotion initiatives – some regions experienced a decline. Importantly, 77.4% of individuals aged 65 years and older were still found to have inadequate or problematic health literacy [25]. These findings highlight the critical need for targeted interventions aimed at improving health literacy, particularly among older adults and in regions with persistent disparities.

## Strengths and limitations

A key strength of this study is the use of nationally representative data from the Turkey Older Persons Profile Survey 2023, which provides a robust basis for generalizing the findings to the older adult population in Turkey. The large sample size and inclusion of a comprehensive set of sociodemographic, health-related, behavioural, and healthcare access variables allowed for a multidimensional analysis of vaccination determinants. Moreover, the application of LASSO regression for variable selection helped mitigate issues related to multicollinearity and overfitting, ensuring a more parsimonious and interpretable final model.

However, the study also has limitations. First, the cross-sectional design precludes any inference of causality between the identified factors and vaccination status. Second, influenza vaccination status was self-reported, which may introduce recall bias or misclassification. Lastly, while a wide range of covariates were examined, some potentially relevant factors – such as vaccine knowledge, vaccine-related beliefs, trust in the healthcare system, or physician recommendation – were not captured in the survey, as the data were not specifically collected for the purpose of this study.

## Conclusion

This study highlights that influenza vaccination uptake among older adults in Turkey remains insufficient, despite being freely available. Increasing engagement with the healthcare system among older individuals – particularly by improving the utilization of primary care services and promoting the use of mobile health applications – may enhance vaccination rates. Priority should be given to targeted outreach efforts aimed at socially disadvantaged populations, individuals not benefiting from General Health Insurance, and those residing in underperforming regions. These strategies may serve as key interventions to improve influenza vaccine coverage in this vulnerable age group.

## Supporting information

10.1017/S0950268826101563.sm001 Tozduman and Gülle supplementary material Tozduman and Gülle supplementary material

## Data Availability

The data that support the findings of this study are available upon request from TurkStat.

## Supplementary material

The supplementary material for this article can be found at http://doi.org/10.1017/S0950268826101563.

## References

[1] WHO (2025) Influenza (seasonal). WHO fact sheet on influenza. https://www.who.int/news-room/fact-sheets/detail/influenza-(seasonal) (accessed 28 April 2025)

[2] TurkStat (2025) Elderly Statistics. https://data.tuik.gov.tr/Bulten/Index?p=Istatistiklerle-Yaslilar-2024-54079 (accessed 28 May 2025)

[3] Kizmaz M, et al. (2020) Influenza, pneumococcal and herpes zoster vaccination rates among patients over 65 years of age, related factors, and their knowledge and attitudes. Aging Clinical and Experimental Research 32(11), 2383–2391. 10.1007/s40520-019-01423-z. 31776859

[4] Guclu OA, et al. (2019) Relationship of pneumococcal and influenza vaccination frequency with health literacy in the rural population in Turkey. Vaccine 37(44), 6617–6623. 10.1016/j.vaccine.2019.09.049. 31542263

[5] Mutlu H, Coşkun F and Sargin M (2018) The incidence and awareness of vaccination among people aged 65 and over applied to a family medicine outpatient clinic. Ankara Medical Journal, 18. 10.17098/amj.408968.

[6] Sezerol MA and Davun S (2023) COVID-19 vaccine booster dose acceptance among older adults. Vaccine 11(3), 3. 10.3390/vaccines11030542. PMC10056148 36992126

[7] Melike Mercan Başpınar ET (2020) A comparison of the seasonal influenza vaccination rates and related factors. The Medical Bulletin of Haseki. Published online June 17. 10.4274/haseki.galenos.2020.5979.

[8] Sofuoglu RS, Di̇Bek Büyükdi̇Nç M and Başak O (2024) Vaccination frequency and associated factors in older adults: A primary care-based cross-sectional study. Turkish journal of Geriatrics 27(1), 31–41. 10.29400/tjgeri.2024.376.

[9] Kahraman H (2024) Erişkin Bağışıklamada Kaçan Fırsatlar. Osmangazi Journal of Medicine 46(4). 10.20515/otd.1491532.

[10] Dönmez AÇ, Güzel EÇ and Topçu B. (2024) Investigation of pneumococcus, influenza, Covid-19 vaccination rates and affecting factors in patients aged 65 and over. Konuralp Medical Journal. 16(2), 2. 10.18521/ktd.1361094.

[11] Medetalibeyoğlu A and Ezirmik E (2020) A study on determining the level of knowledge about influenza, pneumococcal, herpes zoster, and tetanus vaccines among the vaccines recommended by the World Health Organization and the level of vaccination in individuals sixty-five years old and over. The Medical Bulletin of Haseki 58(4), 414–421.

[12] Dereli F, et al. (2022) 65 Yaş ve Üzeri Bireylerin Bağışıklama Durumlarının Belirlenmesi: Aile Sağlığı Merkezi Örneği. İzmir Katip Çelebi Üniversitesi Sağlık Bilimleri Fakültesi Dergisi 7(2), 2.

[13] Yalçın Gürsoy M, et al. (2022) Vaccination coverage and related factors among the elderly: A cross-sectional study from Turkey. Public Health Nursing. 39(2), 390–397. 10.1111/phn.12972. 34551144

[14] TurkStat (2023) Türkiye Older Persons Profile Survey. https://data.tuik.gov.tr/Bulten/Index?p=Turkiye-Yasli-Profili-Arastirmasi-2023-53809 (accessed 21 May 2025).

[15] Jemna DV, et al. (2024) Socio-economic inequalities in the use of flu vaccination in Europe: A multilevel approach. Health Economics Review. 14(1), 61. 10.1186/s13561-024-00535-1. 39083186 PMC11292999

[16] Roller-Wirnsberger R, et al. (2021) The role of health determinants in the influenza vaccination uptake among older adults (65+): A scope review. Aging Clinical and Experimental Research 33(8), 2123–2132. 10.1007/s40520-021-01793-3. 33587270 PMC7882864

[17] Okoli GN, et al. (2020) Seasonal influenza vaccination in older people: A systematic review and meta-analysis of the determining factors. PLoS One 15(6), e0234702. 10.1371/journal.pone.0234702. 32555628 PMC7302695

[18] Rudvan Al Lİ, Sönmezer MÇ and Ünal S (2021) Where are we in adult vaccination? Evaluation to vaccination status of adults aged 65 and over who applied to the adult immunization unit of a tertiary University Hospital in Turkey. Ankara Medical Journal. 21(3), 350–363. 10.5505/amj.2021.67778.

[19] Zaraket H, et al. (2019) Review of seasonal influenza vaccination in the eastern Mediterranean region: Policies, use and barriers. Journal of Infection and Public Health. 12(4), 472–478. 10.1016/j.jiph.2018.10.009. 30446255

[20] Sayaca N (2023) Pneumococcal and influenza vaccine awareness in individuals over 65 years. Anatolian Current Medical Journal. 5(4), 398–404. 10.38053/acmj.1344692.

[21] Social Security Institution (2025) What is General Health Insurance?. https://www.sgk.gov.tr/Content/Post/742c02df-68e1-422c-a387-fa2e4326b015/Genel-Saglik-Sigortasi-nedir-2023-01-25-11-25-46 (accessed 27 May 2025).

[22] Harris JA, et al. (2016) Obesity and the receipt of influenza and pneumococcal vaccination: A systematic review and meta-analysis. BMC Obesity. 3(1), 24. 10.1186/s40608-016-0105-5. 27200179 PMC4855336

[23] Kan T and Zhang J (2018) Factors influencing seasonal influenza vaccination behaviour among elderly people: A systematic review. Public Health 156, 67–78. 10.1016/j.puhe.2017.12.007. 29408191 PMC7111770

[24] Ilhan MN, et al. (2016) Prevalence and socio-demographic determinants of tobacco, alcohol, substance use and drug misuse in general population in Turkey. Noro Psikiyatri Arsivi. 53(3), 205–212. 10.5152/npa.2015.10050. 28373796 PMC5378212

[25] Ministry of Health of Turkiye, Directorate General for Health Promotion (2024) Surv ey on Health Literacy Levels and Associated Factors in Turkiye (Publication No. 1298). https://dosyamerkezi.saglik.gov.tr/Eklenti/50280/0/turkiye-saglik-okuryazarligi-ve-iliskili-faktorleriarastirmasipdf.pdf (accessed 20 May 2025).

