# Supplementary material for: Influenza vaccination coverage and determinants of vaccination among older adults in Turkey
Source: Epidemiol Infect. 2026 Apr 30;154:e59. doi: 10.1017/S0950268826101563 (PMC13177008; doi:10.1017/S0950268826101563)
Supplement: Tozduman and Gülle supplementary material [file S0950268826101563sup001.docx]

Table S1. Health-Related Characteristics by Vaccination Status

|  |  | Vaccinated  (n=2,266) | Not vaccinated (n=9,391) | Total |
| --- | --- | --- | --- | --- |
| General health | Very good | 27 (18.5%) | 119 (81.5%) | 146 |
| status | Good | 422 (17.4%) | 2,004 (82.6%) | 2,426 |
|  | Moderate | 1,200 (21.2%) | 4,473 (78.8%) | 5,673 |
|  | Bad | 542 (18.5%) | 2,394 (81.5%) | 2,936 |
|  | Very Bad | 75 (15.8%) | 401 (84.2%) | 476 |
| Chronic disease | Yes | 1,921 (20.9%) | 7,263 (79.1%) | 9,184 |
| Officially documented disability | Yes | 184 (18.1%) | 835 (81.9%) | 1,019 |
| Regular prescribed medication use | Yes | 1,987 (21.4%) | 7,301 (78.6%) | 9,288 |
| Tobacco | Yes, every day | 235 (18.1%) | 1,065 (81.9%) | 1,300 |
| consumption | Yes, occasionally | 45 (18.4%) | 200 (81.6%) | 245 |
|  | No, I used to smoke | 662 (26.7%) | 1,817 (73.3%) | 2,479 |
|  | No, never smoked | 1,324 (17.3%) | 6,309 (82.7%) | 7,633 |
| Ever consuming alcohol | Yes | 420 (31.7%) | 907 (68.3%) | 1,327 |
| Physical activity | Never | 1,037 (17.0%) | 5,074 (83.0%) | 6,111 |
|  | Rarely | 338 (17.3%) | 1,620 (82.7%) | 1,958 |
|  | 1-3 times a month | 97 (24.9%) | 292 (75.1%) | 389 |
|  | At least once a week | 277 (23.6%) | 896 (76.4%) | 1,173 |
|  | Almost every day | 517 (25.5%) | 1,509 (74.5%) | 2,026 |
| The Washington Group Short Set on Functioning | At least one functional limitation | 861 (17.8%) | 3,973 (82.2%) | 4,834 |
| First consulted  health institution | Family health centre | 1,103 (21.7%) | 3,982 (78.3%) | 5,085 |
|  | Public hospital | 906 (16.6%) | 4,558 (83.4%) | 5,464 |
|  | University hospital | 59 (21.3%) | 218 (78.7%) | 277 |
|  | Private hospital | 124 (25.6%) | 361 (74.4%) | 485 |
|  | Private polyclinic/medical centre | 6 (46.2%) | 7 (53.8%) | 13 |
|  | Private clinic | 0 (0.0%) | 11 (100.0%) | 11 |
|  | City hospital | 64 (22.0%) | 227 (78.0%) | 291 |
|  | Traditional methods/complementary medicine (cupping, folk healer, reiki, etc.) | 1 (11.1%) | 8 (88.9%) | 9 |
|  | Home healthcare | 3 (13.6%) | 19 (86.4%) | 22 |
| Have difficulties  with | Transportation to a health institution | 669 (17.9%) | 3,071 (82.1%) | 3,740 |
|  | Communication with health professionals | 361 (18.0%) | 1,641 (82.0%) | 2,002 |
|  | Making an appointment | 1,160 (20.1%) | 4,617 (79.9%) | 5,777 |
|  | Accessing a preferred physician | 734 (21.1%) | 2,737 (78.9%) | 3,471 |
|  | Doing paperwork during healthcare | 511 (18.4%) | 2,260 (81.6%) | 2,771 |
|  | Waiting in line | 751 (20.3%) | 2,945 (79.7%) | 3,696 |
|  | Limited examination periods | 588 (21.0%) | 2,207 (79.0%) | 2,795 |
|  | The process of taking medication | 226 (16.9%) | 1,108 (83.1%) | 1,334 |
|  | Physically conditions of the health institution | 350 (18.2%) | 1,570 (81.8%) | 1,920 |
| Received home healthcare in the last 12 months? | Yes | 63 (16.6%) | 316 (83.4%) | 379 |
| mHealth usage | Yes | 467 (29.8%) | 1,098 (70.2%) | 1,565 |
|  | |  |  | mean±S.D. |
| Lawton-Brody Instrumental Activities of Daily Living Scale | | 6.20±2.19 | 5.60±2.48 | 5.72±2.44 |
| Katz Index of Independence in Activities of Daily Living | | 5.56±1.15 | 5.36±1.50 | 5.4±1.44 |

Table S2. Determinants for Influenza Vaccination-Binary Logistic Regression

|  |  | O.R. (95% CI) |
| --- | --- | --- |
| Age | | 1.04 (1.03-1.05) |
| BMI | | 1.01 (1-1.03) |
| Lawton-Brody Instrumental Activities of Daily Living Scale | | 1.06 (1.03-1.1) |
| Marital status | Married | 1.49 (0.92-2.53) |
| (ref: Never | Divorced | 1.06 (0.61-1.93) |
| married) | Widowed | 1.18 (0.73-2.02) |
| Education | No school completed | 1.53 (1.25-1.87) |
| (ref: Illiterate) | Primary school | 1.52 (1.3-1.78) |
|  | General secondary school/vocational or technical secondary school/primary education | 1.8 (1.41-2.29) |
|  | General high school/ vocational or technical high school | 1.87 (1.48-2.35) |
|  | 2 or 3 year higher educational institutions/ 4 years universities or other higher educational institutions | 2.34 (1.84-2.97) |
|  | 5 or 6 years faculties/ Master (Except for 5 or 6 years faculties)/ Doctorate | 3.32 (2.03-5.39) |
| Employment | Employed | 1.09 (0.86-1.38) |
| status | Retired or left work life due to reasons related to age | 1.11 (0.9-1.37) |
| (ref:Not retired | Disabled and/or unable to work due to permanent health problems | 1.26 (0.93-1.7) |
| nor employed) | Occupied with housework and/or with the care of children, elderly, ill etc. people in family | 1.1 (0.91-1.33) |
| Income | Very easy | 2.15 (1.39-3.29) |
| sufficiency | Easy | 0.83 (0.66-1.04) |
| (ref: Very hard) | Moderate | 1.02 (0.85-1.23) |
|  | Hard | 0.98 (0.82-1.19) |
|  | No answer | 1.05 (0.86-1.28) |
| Have Social security coverage | Yes | 1.42 (1.17-1.74) |
| General health | Very good | 0.86 (0.5-1.46) |
| Status (ref: Very | Good | 0.83 (0.61-1.14) |
| bad) | Moderate | 0.97 (0.73-1.3) |
|  | Bad | 1.06 (0.8-1.42) |
| Chronic disease | Yes | 1.15 (0.97-1.37) |
| Regular prescribed medication use | Yes | 1.74 (1.45-2.09) |
| First consulted | Family health centre | 1.02 (0.82-1.29) |
| health institution | Public hospital | 0.87 (0.7-1.1) |
| (ref: private | University hospital | 0.88 (0.6-1.27) |
| healthcare services) | City hospital | 1.04 (0.73-1.49) |
| Have difficulties | Accessing a preferred physician (no/yes) | 0.92 (0.81-1.04) |
| with | Accessing a preferred physician (no/irrelevant) | 0.54 (0.27-1.02) |
|  | Waiting in line (no/yes) | 0.93 (0.81-1.07) |
|  | Waiting in line (no/irrelevant) | 1.42 (0.56-3.55) |
|  | Limited examination periods (no/yes) | 0.96 (0.83-1.11) |
|  | Limited examination periods (no/irrelevant) | 0.97 (0.46-1.9) |
|  | Physically conditions of the health institution (no/yes) | 0.94 (0.81-1.09) |
|  | Physically conditions of the health institution (no/irrelevant) | 0.57 (0.27-1.11) |
| Tobacco | Occasionally | 1.02 (0.7-1.46) |
| Consumption (ref: | Former smoker | 1.41 (1.18-1.68) |
| daily smoker) | Never smoked | 1.11 (0.94-1.33) |
| Ever consuming alcohol (ref: No) |  | 1.41 (1.21-1.64) |
| Physical activity | Rarely | 0.89 (0.77-1.03) |
| (ref: never) | 1-3 times a month | 1.36 (1.05-1.74) |
|  | At least once a week | 1.11 (0.94-1.3) |
|  | Almost every day | 1.26 (1.09-1.45) |
| mHealth usage (ref:no) | Yes | 1.23 (1.05-1.43) |
|  | Not applicable | 0.7 (0.58-0.83) |
| NUTS-1 Region | TR1: İstanbul | 1.68 (1.26-2.26) |
| (ref: TRB: Central | TR2: West Marmara | 1.38 (1.02-1.88) |
| East Anatolia) | TR3: Aegean | 1.65 (1.24-2.2) |
|  | TR4: East Marmara | 1.48 (1.11-2.01) |
|  | TR5: West Anatolia | 2.06 (1.54-2.77) |
|  | TR6: Mediterranean | 2.02 (1.52-2.7) |
|  | TR7: Central Anatolia | 1.53 (1.11-2.1) |
|  | TR8: West Black Sea | 1.55 (1.15-2.1) |
|  | TR9: East Black Sea | 1.12 (0.81-1.55) |
|  | TRA: North East Anatolia | 1.11 (0.76-1.61) |
|  | TRC: South East Anatolia | 1.61 (1.16-2.24) |

Nagelkerke R²: 0.1089266, Model χ² (61)=822.02, p<0,001, AUC: 0.69 (95% CI: 0.67–0.70, DeLong)
